# Crop Model Parameterisation of Three Important Pearl Millet Varieties for Improved Water Use and Yield Estimation

**DOI:** 10.3390/plants11060806

**Published:** 2022-03-18

**Authors:** Petrus A. Ausiku, John G. Annandale, Joachim Martin Steyn, Andrew J. Sanewe

**Affiliations:** 1 Department of Plant and Soil Sciences, University of Pretoria, Private Bag X20, Pretoria 0028, South Africa; john.annandale@up.ac.za (J.G.A.); martin.steyn@up.ac.za (J.M.S.); adrew.sanewe@gmail.com (A.J.S.); 2 Department of Crop Production and Agriculture Technologies, University of Namibia, Private Bag 13301, Windhoek 9000, Namibia

**Keywords:** crop growth, modelling, extinction coefficient, dry matter production, partitioning, *Pennisetum glaucum*, radiation use efficiency, SWB

## Abstract

Pearl millet is an important crop for food security in Asia and Africa’s arid and semi-arid regions. It is widely grown as a staple cereal grain for human consumption and livestock fodder. Mechanistic crop growth and water balance models are useful to forecast crop production and water use. However, very few studies have been devoted to the development of the model parameters needed for such simulations for pearl millet. The objectives of the study were to determine crop-specific model parameters for each of three pearl millet varieties (landrace, hybrid, and improved), as well as to calibrate and validate the Soil Water Balance (SWB) model for predicting pearl millet production and water use based on weather data. The SWB was chosen because it is widely used in southern Africa; however, the developed parameters should benefit other models as well. The presented crop-specific parameter values were derived from field observations and literature. Varieties with different phenology, maturity dates and tillering habits were grown under well-watered and well-fertilised conditions for calibration purposes. The calibrated model was used to predict biomass production, grain yield and crop water use. The hybrid’s water use efficiency was higher than that of the landrace and improved variety.

## 1. Introduction

Pearl millet (*Pennisetum glaucum* (L.) R.Br.) is an important crop in subsistence agriculture in large areas of the semi-arid tropics of Africa and India, where it is widely grown for grain, fodder and fuel [1,2,3]. In terms of annual production, pearl millet is the sixth most important cereal crop in the world [4,5]. In southern Africa, pearl millet is mainly cultivated in Botswana, Namibia, Zambia, Zimbabwe and South Africa [6]. People in northern Namibia are almost entirely dependent on pearl millet for food [7]. Although pearl millet is widely cultivated by resource-poor farmers as a traditional staple grain crop, it is not as well-known as a major grain crop outside subsistence agriculture. Relatively little is grown commercially as it is mostly used as a fodder crop by commercial farmers. Pearl millets are known for their high levels of resilience to climate change effects, and good nutritional properties [8]. Pearl millets, therefore, play a critical role in improving food security for resource-poor farmers. *Pennisetum glaucum*, a C4 grass, has excellent photosynthetic efficiency and biomass production potential. Millet grain is highly nutritious, with 8–19% protein, low starch, high fibre (1.2%) [9], and higher micronutrient concentrations (iron and zinc) than rice, wheat, maize and sorghum [10]. Despite the clear importance of this crop, pearl millet is mainly grown under dryland conditions, characterised by marginal production environments, and with minimal use of commercial inputs, such as adequate irrigation and fertilisers. Pearl millet genotypes differ greatly in growth pattern and plant structure and generally require irrigation for optimum yield [11,12,13,14].

Recently, models have been extensively used to predict future events for different scenarios [15] and provide information on resource dynamics at individual sites, over regions and the globe [16,17]. According to Kasampalis et al. [18], models play a crucial role in the development of sustainable land management strategies for maximising the sustainability goals of land managers and policymakers. Models fall into two general categories: empirical and more mechanistic [19]. The empirical models attempt to portray observed phenomena without hypothesising how they occurred. Mechanistic models, on the other hand, seek to describe cause and effect. Mechanistic models, therefore, require understanding and mathematical descriptions of the underlying processes [20,21].

Crop models have become increasingly useful for different purposes, primarily in education and technology transfer [22,23], decision making, and as agronomic research tools to predict crop growth and development, reduce yield gaps, increase the understanding of water management and support interpretation of experimental results [24,25,26,27]. Crop growth models are important tools for scientific research and are widely applied in agriculture to make predictions about the agronomic, environmental, and economic consequences of the complex interactions between crop management, soil and weather conditions [28,29,30,31,32,33,34,35]. Crop models, along with short-term field experiments, are developed as tools to assess yield losses associated with the effects of climate change, changes in temperature, environmental stresses, poor crop management and rainfall on crop yield. Moreover, lengthy and expensive field experiments, particularly with a high number of treatments, can be pre-evaluated through a well-proven model to improve field tests and to lower their overall costs [36]. There are many models, and they all need either crop-specific or even variety-specific model parameters, which are often not available, but need to be determined from growth analysis and water balance studies. These parameters help to project future crop growth and yields for different regions, irrigation demand and to understand the effect of crop and soil type on food productivity and future soil fertility [37,38].

Crop growth simulation models have been used to investigate the effects of crop management options such as irrigation timing, amount and fertiliser applications, under different environmental conditions on long-term mean yield [39,40,41,42]. For the models to simulate yields across the full range of possible yields, it must be robust, thus it is important to evaluate model performance under high-yield conditions in which yields approach the yield potential ceiling, as well as in environments that produce lower yield levels under stress. The projection of crop yield potential, the attainable yield and the corresponding yield gap, is vital to assist in meeting the challenge of increasing food production demands of a growing world population [43]. This necessitates an in-depth understanding of crop development and growth, which is influenced by a variety of climatic, edaphic, hydrological, physiological and managerial factors. Agricultural yields are well below attainable levels in many parts of the world and closing yield gaps is widely viewed as an important strategy to secure a sufficient and reliable food supply [38,44]. Fundamentally, yield gaps are caused by deficiencies in biophysical crop growth environments that are not addressed by agricultural management practices [45]. Crop models can be used to estimate potential yields for a site based on weather conditions and soil water-holding characteristics of the site, and then a systems approach can be taken to determine causes of, and possible remedies for minimising the yield gap between the potential and the actual yields. A good understanding of crop water use is needed to optimise irrigation water management at the field level, for irrigation system design, and to influence water and energy savings [46,47]. Field-scale crop models have been subject to a broad range of uncertainty analyses, varying from grain crop yield response to biomass performance [48].

This paper specifically looked into the Soil Water Balance (SWB) model because it is widely used in southern Africa. The SWB model is a mechanistic crop growth model that is well established and widely used [46,47,49,50,51,52,53,54]. SWB is designed to simulate crop growth, water use and soil water balance components from weather, soil and crop data. Evapotranspiration (ET) is calculated from weather data using Penman–Monteith grass reference daily evapotranspiration (ETo) [55]. Soil water movement is simulated through a layered cascading model, where each layer can have its particular physical properties [22]. Precipitation interception, runoff and drainage are also accounted for in the model. Dry mass production is calculated daily from crop emergence to maturity as either transpiration or radiation-limited [56], whereas assimilates are partitioned to distinctive plant organs, depending on the phenological stage determined from thermal time and water stress level. Robust crop parameters are needed to successfully apply models and should be obtained from careful measurements under field conditions [57]. Some model parameters have already been published for several important cultivated crops [58,59,60,61], but there is a lack of crop-specific model parameters for pearl millet varieties. This is particularly true for pearl millet varieties that are grown under irrigation. Therefore, the objectives of this study were to measure growth and water use of improved, hybrid and landrace pearl millet and to derive crop-specific growth parameters for each variety to be used with mechanistic growth simulation models. Furthermore, the parameters are used to calibrate and validate the SWB generic crop model for widely grown varieties in Namibia (Kangara and Kantana) and South Africa (Agrigreen) to enable the establishment of accurate irrigation regimes under well-watered conditions. The model can then also be used to run simulations to predict crop biomass and grain yield response to various precipitation conditions in agricultural fields, which will give better insight into irrigation scheduling and production planning.

## 2. Results

### 2.1. Soil Water Balance

The components of the soil water balance for December 2017–April 2018 for the three pearl millet varieties are shown in Table 1 and Table 2. On 14 December 2017, there was 26 mm of rainfall before germination.

It was observed that volumetric soil water content in the root zone of the landrace variety increased by 21 mm from 328 to 350 mm. Similar trends were observed in both hybrid and improved varieties during the same period. During the seedling stage, there was an increase in soil water content due to irrigation and rainfall.

Seasonal ET varied between 598 and 670 mm (Table 2) and varied between 7 and 89 mm for weekly ET (Table 2). The total water input (rainfall and irrigation) in all plots during the crop seasons (December–April) of 2017/2018 were 947 mm for the landrace, 1035 mm for the improved variety and 1111 mm for the hybrid. Irrigation amount increased with decreasing precipitation and increased as canopy cover increased [62]. About 70 mm of rainfall was received in December, 78 mm in January, 91 mm in February, 230 mm in March and 73 mm in April. Generally, genotypic differences in ET were mostly related to the length of the crop growing period (Table 2).

Higher drainage was observed during the last week of March. In all varieties this was due to a high rainfall event. In March about 208 mm of rain fell during the late growth period, concentrated mainly in three days, when the soil was wet from previous irrigations, and while water consumption by the crop was low. The amount of drainage below the root zone (1 m soil depth) varied from 0 to 88 mm during growing seasons. The total drainage observed in the landrace was higher than improved and hybrid. Total drainage value represents 18.9% (landrace), 14.7% (improved) and 13.4% (hybrid) of the water applied by irrigation and rainfall.

#### 2.1.1. Radiation Limited Dry Matter Production

**Figure 1:**
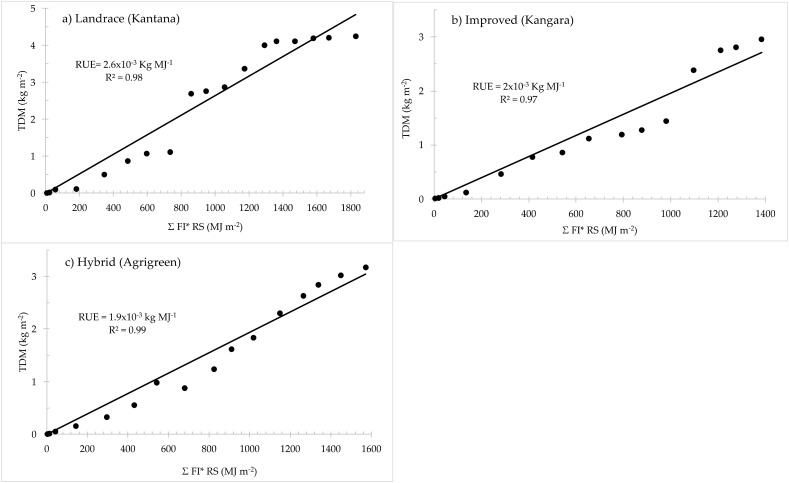
Cumulative top dry matter (TDM) production of the three pearl millet varieties: (**a**) landrace, (**b**) improved and (**c**) hybrid as a function of the cumulative (∑) product of fractional interception and solar radiation (FI × Rs). Radiation use efficiency (RUE) and the coefficient of determination (R^2^) are shown. FI values were determined on the day of measurement.

The radiation use efficiency (RUE) is the slope of the relationship between the total dry matter produced and cumulative intercepted radiation. High R^2^ values (>0.97) were achieved for all three pearl millet varieties (Figure 1), highlighting the very robust positive relationship between these two variables. The calculated RUE was 2.6 × 10^−3^ kg MJ^−1^ for the landrace, 1.9 × 10^−3^ kg MJ^−1^ for the hybrid and 2 × 10^−3^ kg MJ^−1^ for the improved variety grown under optimal conditions.

The values for both the hybrid and improved variety compares well with those found in the literature for pearl millet (1.96 × 10^−3^ kg MJ^−1^ [63], 2.0 × 10^−3^ kg MJ^−1^ [64] and 2.1 ± 2.4 × 10^−3^ kg MJ^−1^ [65,66]). Similarly, the higher RUE calculated for the landrace is just above that reported by Ong and Monteith [67] (2.5 × 10^−3^ kg MJ^−1^) but well below the 4 × 10^−3^ kg MJ^−1^ found by Ram et al. [68]. The calculated radiation extinction coefficients (Ks) for the three pearl millet varieties vary from 0.32 to 0.53. These values are closely related to those for field measurements taken at noon reported in the literature (0.29 [69] and 0.5 [67]). The landrace had lower Ks due to its vertical and clumped leaves, which allowed more solar radiation to penetrate through the canopy, leading to more dry matter production [5].

#### 2.1.2. Water Limited Dry Matter Production

Dry matter water ratio (DWR) values determined for the three varieties are presented in Table 3. The hybrid (Agrigreen) had substantially higher water use efficiency characteristics compared to the improved and landrace varieties for grain. The value of WUE (9.16 kgm^−3^) for landrace biomass was in the range of that reported by Payne [69].

**Table 3:** Measured crop-specific growth parameters for three pearl millet varieties: Hybrid (Agrigreen), landrace (Kantana) and improved (Kangara) and other values were sourced from literature *.

Crop Parameter	Value			Literature Value	Reference
	Hybrid (Agrigreen)	Landrace (Kantana)	Improved (Kangara)		
Canopy extinction coefficient for photosynthetic active radiation K_PAR_	0.43	0.40	0.42	0.64–0.42	[70,71]
Canopy extinction coefficient for total solar radiation Ks	0.31	0.28	0.30	0.49	[72]
Radiation use efficiency, RUE (kg MJ^−1^)	0.0019	0.0026	0.002	0.0003–0.00261	[64,65,73,74,75,76]
Dry matter/transpiration ratio corrected for vapour pressure deficit, DWR (Pa)	11.3	15.8	11.2		
Water use efficiency_grain,_ WUE (kg m^−3^)	1.43	1.29	1.23	3.16–10.4	[11,12]
Water use efficiency_biomass,_ WUE (kg m^−3^)	4.83	9.16	4.69	8.56–39.6	[11,12]
Specific leaf area, SLA (m^2^ kg^−1^)	22.49	19.91	23.34	11.98–33	[77,78,79]
Leaf-stem partition parameter, PART (m^2^ kg^−1^)	2.76	1.27	2.48		
Maximum root depth (m)	1.00	1.00	1.00	1.8	[80,81,82]
Maximum crop height (m)	2.87	4.22	2.80	4.9	[6]
Maximum transpiration rate (mm d^−1^)	9 *	9 *	9 *	9.2	[83]
Base temperature (°C)	10 *	10 *	10 *	10–12	[67,84,85]
Optimum temperature (°C)	33 *	33 *	33 *	33–34	[67,84]
Maximum temperature (°C)	45 *	45 *	45 *	45–47	[67,84,85]
Emergence day degrees (°C d)	60	64	60	60	[67]
Flowering day degrees (°C d)	900	1058	832	954–1265	[84]
Maturity day degrees (°C d)	1686	2124	1480	1552–1714	[84]
Transition day degrees (°C d)	670	780	655	415–621	[84]
Total dry matter yield at emergence (kg m^−2^)	0.0019	0.0019	0.0019		
Stress index	0.30 *	0.30 *	0.30 *	0.30–0.50	[86]

Values with superscript star (*) are adopted from literature.

The grain yield WUE of the hybrid (1.43 kgm^−3^) was higher than that recorded for both the landrace (1.29 kgm^−3^) and improved (1.23 kgm^−3^) variety, but lower than the seasonal average determined by Payne [69].

#### 2.1.3. Growing Degree Days

Growing degree days (GDD) are widely used for describing the developmental response of crops to temperature. GDDs recorded from emergence until senescence for the three varieties ranged between 1480–2124 day degrees (Table 3). All three of the pearl millet varieties had similar emergence GDD. The improved variety needed the lowest thermal time to flowering and maturity., The improved (Kangara) pearl millet variety reached flowering after 832 °C d, while for the landrace, the flowering stage was reached at 1058 °C d and the hybrid required 900 °C d to flower. Similar findings were also reported by Singh et al. [84]. The variation in GDDs is due to differences in developmental rates among the three varieties.

#### 2.1.4. Radiation Interception

Maximum fractional interception of radiation of the three pearl millet varieties was almost identical and reached a maximum of 0.94–0.98, similar to what was reported by Squire et al. [65]. The highest K_PAR_ for the hybrid pearl millet variety was 0.43, with an LAI of 6.79 which intercepted 96% of solar radiation. The landrace and improved variety recorded a lower K_PAR_ value than for the hybrid. The extinction coefficient for solar radiation (Ks) was 0.30 for the improved pearl millet variety.

**Figure 2:**
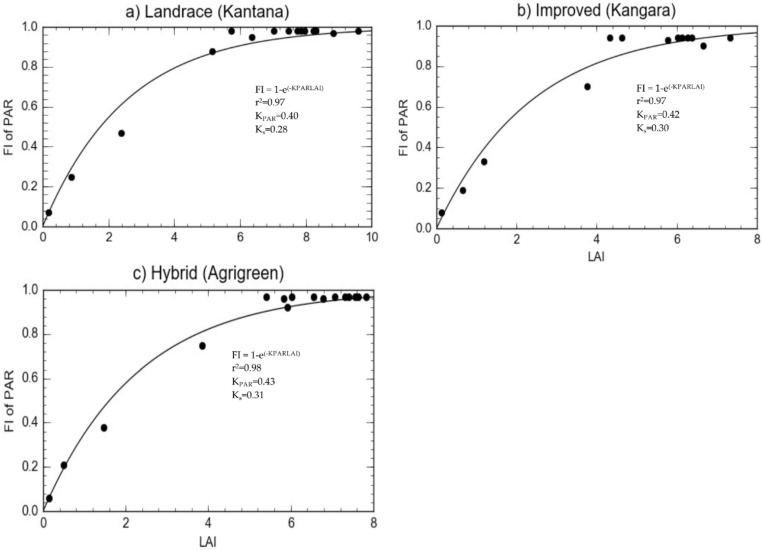
The relationship between leaf area index (LAI) and fractional interception (FI) of photosynthetically active radiation (PAR) for three pearl millet varieties: (**a**) landrace, (**b**) improved and (**c**) hybrid. The coefficient of determination (R^2^), as well as the canopy extinction coefficient for photosynthetically active radiation (K_PAR_) and total solar radiation (K_s_) are shown.

The radiation interception and canopy LAI varied from planting to harvesting and showed a similar trend to those observed by other authors [84]. The LAI (9.59) and FI_PAR_ (98%) were generally higher for the landrace at the heading stage. Similarly, the improved variety recorded its highest LAI (6.99) and FI_PAR_ (94%) also at heading. However, for the hybrid, the greatest LAI and FI_PAR_ were observed at grain filling. Calculated Ks values for improved, landrace and hybrid varieties for well-watered treatments are reported in Table 3 and Figure 2.

#### 2.1.5. Above-Ground Biomass Partitioning

**Figure 3:**
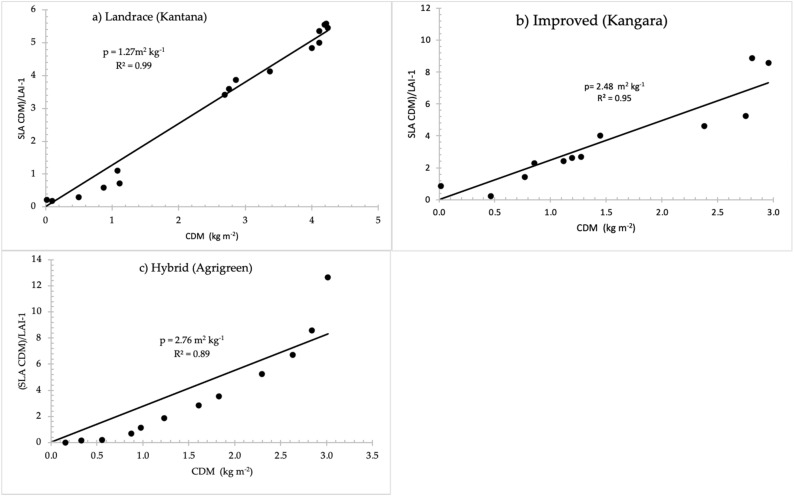
Determination of the stem-leaf dry matter partitioning parameter (p) as a function of canopy dry matter (CDM), specific leaf area (SLA) and leaf area index (LAI) for pearl millet varieties: (**a**) landrace, (**b**) improved and (**c**) hybrid, with the slope of the regression line (p) and the coefficient of determination (R^2^).

Partitioning of total dry matter to different plant parts was observed to differ between varieties. For example, the leaf to stem (stem + leaf sheath) partition parameter was 2.76 m^2^ kg^−1^ for the hybrid, 2.48 m^2^ kg^−1^ for the improved variety and 1.27 m^2^ kg^−1^ for the landrace (Figure 3 and Table 3). The specific leaf area (SLA) and the leaf-stem dry matter partitioning parameter (p) have to be known to compute DM partitioning with SWB [46]. The dry matter produced is partitioned among roots, leaves, stems and grain. Partitioning depends on the phenological development stage of the crop, as well as water stress [87]. Figure 3 illustrates the correlation between canopy dry matter (CDM) and (SLA CDM)/LAI-1 for each of the three pearl millet varieties. The slope of the regression line, which is forced through the origin, represents p in m^2^ kg^−1^. Values of p for the three pearl millet varieties are summarised in Table 3.

#### 2.1.6. Specific Leaf Area

**Figure 4:**
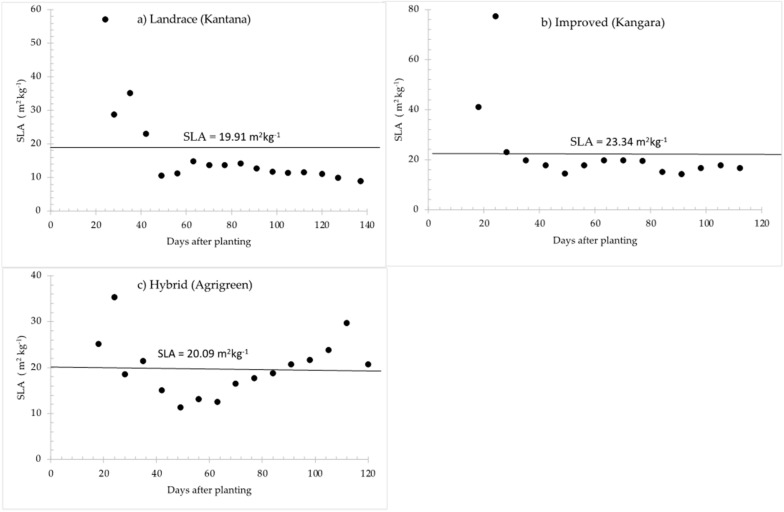
Measured values of specific leaf area (SLA) during the growing season of three pearl millet varieties: (**a**) landrace, (**b**) improved and (**c**) hybrid, the value shown on horizontal line is the average SLA for each variety.

The values of SLA presented in Table 3 and Figure 4 are averages obtained over the entire growing season. The average values recorded for SLA were 22.49 m^2^ kg^−1^ for the hybrid, 19.91 m^2^ kg^−1^ for the landrace and 23.34 m^2^ kg^−1^ for the improved variety. Specific leaf area (SLA) is vital in leaf and plant functioning and regulates the strategy of resource acquisition and plant growth [88]. SLA is presented in Figure 4 as a mean of all leaves measured over the growing season. The measured SLA (m^2^ kg^–1^) ranged between 19.91 and 23.34 m^2^ kg^−1^, which are in accordance with values of 18 and 33 m^2^ kg^−1^ reported by Singh [77], Van Heemst [78] and Monteith [79]. It is clear that SLA is higher at the beginning of the season and the use of a single value for this parameter in SWB is perhaps not ideal.

### 2.2. Model Calibration

The parameters derived from the calibration data set were used to calibrate the SWB model for each of the three pearl millet varieties (Table 3). Field measurements of LAI, FI_PAR_, biomass produced, grain yield and measured soil water deficits were used to calibrate the model. Root depth was not measured but estimated from depth of soil water extraction. The crop-specific growth parameters of the three varieties are shown in the list of values in Table 3 under well-watered conditions. Generally, most parameters correspond well to those reported by others [11,12,72] for pearl millet. The three varieties were grown in mean temperature ranges between 13 °C and 32 °C. Pearl millet is mostly cultivated at an optimum temperature of 25 °C [86] to grow and partition new dry matter into different plant parts. Values (1 m) of maximum root depth (RDmax) were generally in the range (0.9 m) of those reported by Gregory and Reddy [82]. The values of the base, optimal and maximum temperatures for pearl millet development were taken from Ong and Monteith [67], Garcia-Huidobro, Monteith, and Squire [85], and Singh, Joshi and Singh [84]. Maximum plant height (Hmax) for the landrace was 4.22 m, while for the hybrid and improved variety, measured Hmax values were 2.87 m and 2.80 m, respectively. On average, the hybrid and improved pearl millet variety were 32% shorter compared to the landrace.

**Figure 5:**
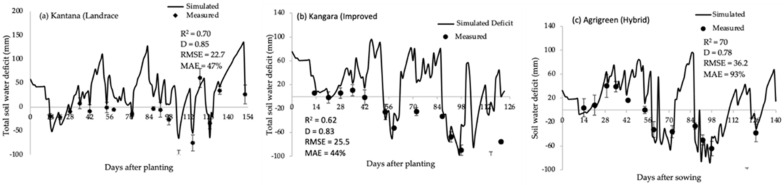
Simulated (solid lines) and measured values (points) of soil water deficits of three pearl millet varieties: (**a**) landrace, (**b**) improved and (**c**) hybrid the value shown on horizontal line is the average total water deficit.

The SWB model was calibrated for the Kantana, Kangara or Agrigreen varieties using the parameters obtained from the calibration data set (Table 3). Calibration was based on field measurements of measured soil water deficits. Soil water deficits were predicted with reasonable accuracy for the calibration data set, with R^2^ = 0.70, D = 0.85 and MAE = 47% for Kantana, R^2^ = 0.62, D = 0.83 and MAE = 44% for Kangara and R^2^ = 0.70, D = 0.78 and MAE = 93% for Agrigreen (Figure 5a–c).

### 2.3. Model Validation

**Figure 6:**
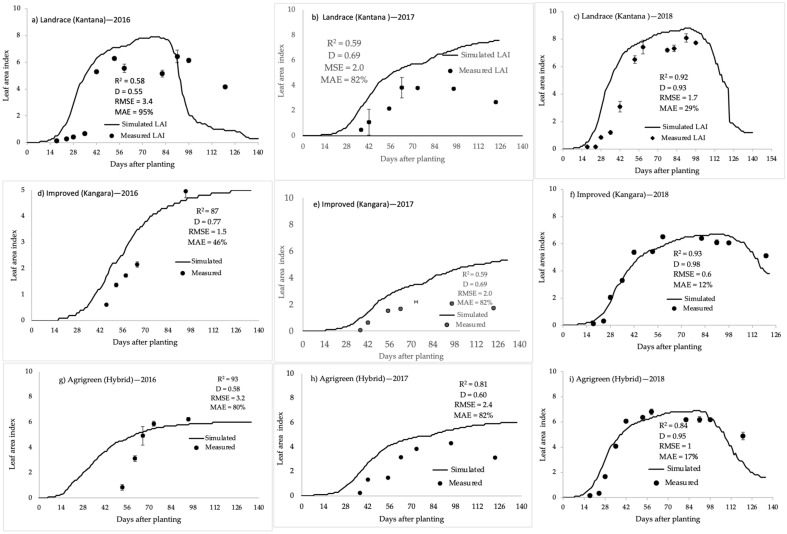
Simulated (solid lines) and measured (points) leaf area index. Landrace (Kantana): (**a**) 2018 rainfed, (**b**) 2017, (**c**) 2018. Improved (Kangara): (**d**) 2016, (**e**) 2017, (**f**) 2018. Hybrid (Agrigreen): (**g**) 2016, (**h**) 2017, (**i**) 2018. All three varieties were irrigated fortnightly excluding (**a**).

SWB validation for leaf area index (LAI) of the varieties grown under different irrigation and weather conditions was obtained from three years of independent experimental data and are presented in Figure 6. LAI was predicted with reasonable to good accuracy for all three varieties grown under a wide range of conditions, giving confidence in the reliability of model output.

**Figure 7:**
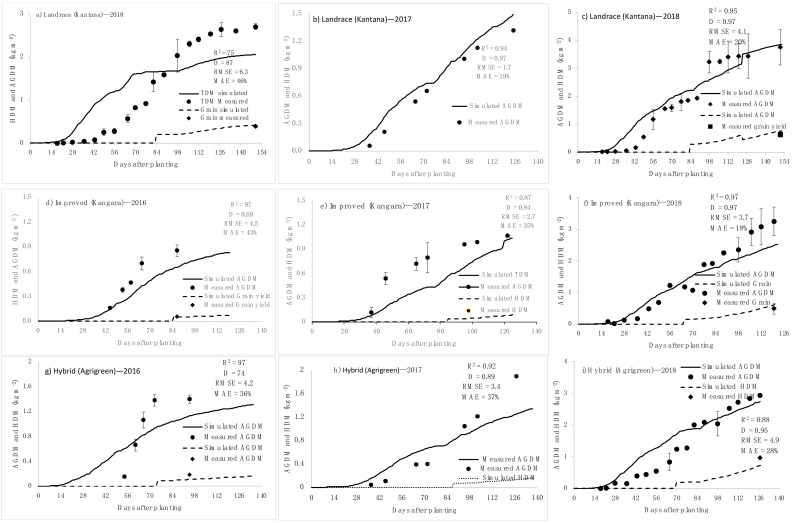
Simulated (solid lines) and measured (points) harvestable dry matter (HDM) and above-ground dry matter (AGDM). Landrace (Kantana): (**a**) 2018 rainfed, (**b**) 2017, (**c**) 2018. Improved (Kangara): (**d**) 2016, (**e**) 2017, (**f**) 2018. Hybrid (Agrigreen): (**g**) 2016, (**h**) 2017, (**i**) 2018. All three varieties were irrigated fortnightly every season excluding (**a**).

The validation of the above-ground dry matter (AGDM) and harvestable dry matter (HDM) yields were predicted with reasonable to good accuracy for pearl millet varieties that were irrigated only once in two weeks (Figure 7c,f,i). Throughout the 2016 to 2017 growing season, the simulated HDM (grain) and AGDM yields followed the same trend as the actual values but were frequently overestimated by the model (Figure 7). The overestimate in 2016 may be due to late planting of the experiment, while the 2017 experiment suffered from frost, resulting in lower actual yield then what is projected by model.

**Figure 8:**
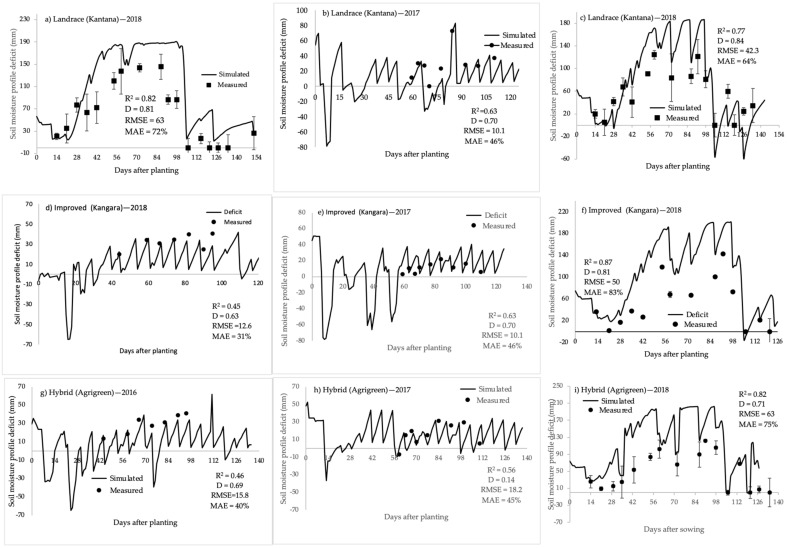
Simulated (solid lines) and measured (points) soil profile deficit to field capacity. Landrace (Kantana): (**a**) 2018 rainfed, (**b**) 2017, (**c**) 2018. Improved (Kangara): (**d**) 2016, (**e**) 2017, (**f**) 2018. Hybrid (Agrigreen): (**g**) 2016, (**h**) 2017, (**i**) 2018. All three varieties were irrigated fortnightly every season excluding (**a**).

The soil water deficits were less accurately estimated, particularly from mid-season onwards, when the crop was rainfed or irrigated less regularly, using the validation data from the 2016–2018 growing season (Figure 8; R^2^ = 0.82, D = 0.72 and MAE = 75% for hybrid; R^2^ = 0.87, D = 0.81 and MAE = 83% for improved; and R^2^ = 0.77, D = 0.84 and MAE = 64% for landrace). These findings imply that the SWB model was able to accurately simulate crop growth, water use and yield for any of the varieties studied under various water supply conditions. As a result, the SWB model can be used with confidence to predict growth, water use and yield of these varieties under various scenarios for future planning.

## 3. Discussion

### 3.1. Soil Water Balance

A total of 542 mm of rain was captured between the date of sowing and harvest, 8% more than the optimum rainfall requirement of pearl millet, indicating that the season was good. The monthly distribution of rainfall in terms of rainy days per month was variable. The seasonal rainfall intensity and monthly distribution, along with cumulative seasonal rainfall variation, cause production uncertainties [89]. During the growing season, ET was different for each of the pearl millet varieties. Seasonal ET for each of the varieties was always less than cumulative precipitation, indicating that soil water supply was not being exhausted. However, at harvest, all three varieties’ seasonal ET was greater than total rainfall, suggesting some extraction of water stored at lower soil depths when no supplemental irrigation was supplied. Under semi-arid conditions, irrigation is one of the most important contributions to ET [90] and this contribution differed depending on the amount of water stored in the soil and crop variety. The water balance data, therefore, suggest an increased risk of exhausting available soil water content due to greater ET, especially for the local landrace and hybrid variety. The greater ET was associated with substantially increased grain yield and above-ground dry matter production. The higher seasonal crop ET recorded for the landrace (670 mm) than the hybrid and improved variety was in part due to late maturity and genotypic differences. In the first two months, no heavy rain occurred early in the growth period, so the crop ET was similar in all varieties.

ET of the landrace was higher due to the increased number of days to maturity. During the seedling stage, there was an increase in soil water content due to irrigation and rainfall. Irrigation amount increased with decreasing precipitation and increased with crop growth [62].

Seasonal irrigation amount was high for the hybrid variety and lower for the landrace. In the first two months, no heavy rain occurred early in the growth period, so the crop ET was similar in all varieties. Higher drainage was observed during the last week of March in all varieties due to a high rainfall event. In March about 208 mm of rain fell during the late growth period, concentrated mainly in three days, when the soil was wet from previous irrigations, and while water consumption by the crop was low. The higher seasonal crop ET recorded for the landrace (670 mm) than the hybrid and improved variety was in part due to late maturity.

### 3.2. Radiation Limited Dry Matter Production

The top dry matter accumulated was high in the landrace variety as it absorbed more radiation over time. This finding is supported by Monteith [66], Sinclair and Muchow [91], who argue that plant growth rate depends on the quantity of radiation intercepted by the canopy which is converted into dry matter accumulation. Further, it was observed among the varieties that the landrace required the highest growing degree days to attain physiological maturity. This is due to the longer growing duration required by the landrace to reach maturity. The RUEs were different for each of the three varieties. According to Monteith [92], Carretero et al. [93], Koester et al. [94] and Teixeira et al. [95], the RUE value depends on the cultivar, water supply, nutrient status, and disease presence. In addition, RUE is rooted in the fundamental relationship between radiant energy use and the accumulation of plant biomass and is influenced by plant phenology and environmental factors [92,96]. Jovanovic et al. [46] also reported that the projected values of RUE denote a lower limit for utilisation of radiation to produce dry matter because root dry matter was excluded in the computations of dry matter for the pearl millet varieties.

Dry matter production is very sensitive to the DWR and moderately sensitive to RDMAX and available water, both of which determine the amount of water used by the plant. Therefore, dry matter production can also be estimated by considering water use efficiency, another important model parameter. In SWB, a simple gas exchange model that acknowledges the tight link between transpiration corrected for dry matter production and vapour pressure deficit (VPD) is used [44]. It was not possible to accurately measure root dry matter, hence this was excluded from the calculation of DWR. Since it was difficult to determine transpiration and total dry matter, ET and above-ground dry matter were used to predict a lower limit value of DWR, after which DWR was adjusted upwards to account for root dry matter yield during model calibration.

### 3.3. Light Interception and Biomass Accumulation

The landrace had lower Ks due to its vertical and clumped leaves, which allowed more solar radiation to penetrate through the canopy, leading to more dry matter production [5]. The improved variety had higher Ks due to its erect leaves, which suggests that PAR was not widely distributed within the canopy, resulting in lower dry matter production. The results are consistent with numerous other studies, which indicate that Ks are correlated with increases in dry matter production as well as final grain yield [6,7,8]. The high RUE observed for the landrace variety could have been associated with the low Ks, as negative correlations between RUE and Ks have been reported for wheat [97] and pigeon pea [98]. The extinction coefficient for Kantana was low (Table 3), suggesting less interception than in both Agrigreen and Kangara. This relationship further suggested that the LAI required to intercept the same amount of PAR was greater in Kantana (landrace) than in Agrigreen (hybrid) and Kangara (improved). The landrace intercepted more PAR than both the hybrid and improved varieties because the landrace had a 20% greater LAI than the improved variety. Pearl millet, a C4 plant, is an efficient crop with a large leaf area index [99]. However, the higher efficiencies were observed due to lower atmospheric saturation vapour pressure deficits of 0.78, 0.83 and 0.82 kPa in Table 3, than other experimental conditions (3.7–5.2 kPa) reported by McIntyre [100]. According to Squire [74], RUE ranged from 1.4 to 2.5 g MJ^−1^ when daily maximum saturation vapour pressure deficits did not exceed 3 kPa. However, lower RUEs (0.3 and 0.8 g MJ^−1^) were reported where daily maximum saturation vapour pressure deficits were between 3 and 5 kPa [74], less than the finding in this study.

Crop genotype influenced fraction of intercepted photosynthetic active radiation (FI_PAR_) and LAI in this study. Photosynthetic active radiation (PAR) is the basic source of energy for dry matter production [101], and LAI followed the pattern of intercepted amount of incoming PAR in the canopy [102]. The landrace had a large amount of foliage which resulted in a higher interception of PAR. This finding is supported by Jonckheere et al. [103] and Huang et al. [4,104], who argued that PAR depends on the amount of foliage represented by LAI, and the orientation and distribution of foliage. More PAR reached the lower canopy in the improved (short-season) pearl millet than in both the landrace and hybrid. This finding is in line with Timlin et al. [105], who reported that short-season varieties produce low biomass and intercept less radiation.

### 3.4. Specific Leaf Area

Specific leaf area (SLA) for all varieties differed during the growing cycles and decreased with time due to the senescence of old leaves. However, a calculated seasonal average value of SLA was used in the SWB model, as supported by Rinaldi [106], that can be used in crop models, without a great source of error in the simulation. SLA has received great attention for accurately simulating the production of assimilates and their distribution to the various plant organs [107]. Both the landrace and hybrid varieties had low SLA and reached leaf senescence later than the improved variety. The SLA values also vary with genotype, modified by weather conditions and factors affecting the plant during the growing period [108,109,110]. Reich et al. [111] reported that SLA is negatively correlated with leaf life span and leaves with low SLA and long life span have lower assimilation rates. The lower SLA of both the hybrid and landrace varieties contributed to long leaf survival, which promotes nutrient retention, enhances long-term photosynthetic rate, nitrogen use efficiency, and protection from desiccation [112,113,114]. The long leaf life span leads to a greater assimilation period, providing the basis for the effective absorption of more solar radiation [115]. The landrace and hybrid also had high leaf weight per leaf area, resulting in low SLA, which is caused by thicker leaves [116]. The high SLA value of the improved variety reduces the amount of assimilation required to produce a given leaf area, which results in an earlier ground cover and consequently a greater light harvest that produces a higher assimilation rate early in the season.

## 4. Materials and Methods

### 4.1. Experiment Description

The field experiments of a detailed growth analysis and calibration of three pearl millet varieties were conducted in an open field at the Hatfield Experimental Farm (25°45′ S, 28°16′ E, 1327 m above sea level) of the University of Pretoria, South Africa. The trial was laid out as a randomised complete block design and treatments were randomly assigned to blocks in all years of the experiment. The field was divided into three blocks with nine plots of 45 m^2^ (5 × 9 m^2^) each in size, with 1 m paths between plots. Plots were demarcated with raised soil bunds between them to avoid the surface movement of water between adjacent plots. The soil was a Hutton sandy clay loam [11] (Soil Classification Working Group, 1991) (loamy, kaolinitic, mesic, Typic Eutrustox) with depths generally in excess of 1.2 m [72]. Nine treatment combinations were set up with three replications. The experiment consisted of three varieties and three irrigation regimes (Table 4). The variety treatments were a hybrid (V_1_: Agrigreen), open pollinated variety (OPV) landrace (V_2_: Kantana) and OPV improved (V_3_: Kangara). The irrigation regimes were well-watered (I_1_: irrigated every week to field capacity), an intermediate irrigation level (I_2_: irrigated every second week to field capacity) and a zero-irrigation control (I_0_: rainfed).

For calibration of the SWB model, the OPV landrace (Kantana), OPV improved (Kangara) and hybrid (Agrigreen) were planted in a well-managed plot. The well-watered plots were used for growth analysis. The pearl millet crops in the experiment were planted at a density of 18 plants per m^2^. For plant growth analysis data were measured from eight plants per plot per sampling event at fourteen days after planting (DAP), thereafter every week until the plants reached physiological maturity. Plant heights and destructive harvests were executed fortnightly for each replicate. The plant samples were separated into leaves, stems and panicles, whereafter the leaf area (LA) was measured using a belt-driven leaf area meter model LI-3100 (LI-COR, Lincoln, NE, USA) calibrated to 0.01 cm^2^. Leaf area at the plant level was calculated as the sum of the areas of each green leaf on a plant, and leaf area index (LAI) was calculated. An electronic balance was used for weighing samples that were oven-dried at 70 °C to constant mass to determine leaf dry matter (LDM) and stem dry matter (SDM). At maturity, grain yield was harvested from 1 m^2^ per plot.

For monitoring the soil water status as part of the soil water balance, a neutron water meter model 503 DR CPN Hydroprobe (Campbell Pacific Nuclear, Martinez, CA, USA) that was calibrated for the experimental site was used to measure soil water contents at 0.2 m increments to a depth of 1.0 m. Root-zone soil water deficit calculations for irrigation were made over an assumed rooting depth of 1.0 m. Weekly soil water content measurements were taken, and thereafter the field was watered to field capacity using a high-density drip system with drip lines spaced 0.45 m apart with an in-line dripper spacing of 0.30 m, and a delivery rate of 8.9 mm h^−1^. The soil water deficit to field capacity was calculated for each layer and the average for the four different layers determined. A profile pit was dug at the experimental site and soil samples were taken at 0.20 m intervals to a depth of 1.0 m to determine bulk density (*ρ_b_*) soil texture and volumetric soil water content (*θ*) at field capacity (FC) and permanent wilting point (PWP). The *ρ_b_* of the soils was determined by gently driving a cylinder of known volume horizontally into the side of the profile pit. The soil was removed and dried at 105 °C for 24 h. Volumetric water content at FC was determined, before planting, by saturating a portion of the field which was then left for 48 h to drain before sampling, while PWP was determined at the end of the season by withholding irrigation on a section of the experimental plot until the plants died. Soil samples of a known volume were taken and *θ* was calculated from the mass loss before and after the soil samples were dried at 105 °C for 48 h. Bulk density varied from 1152–1506 kg·m^−3^ for the 0–1 m soil profile [11].

### 4.2. Soil Water Balance

The water applied to the entire root zone by irrigation or rainfall was used to calculate the change in soil water storage at the end of every week in each plot. The soil water balance method [55,117,118,119] was used for the estimation of evapotranspiration (ET) from the experimental plots. The water balance method is based on the conservation of mass, which states that inputs minus outputs equal the change in soil water content (Δ*S*) of the root zone of a crop, and this is equal to the difference between the water content at end of measurement period *Q_o_*, and the water content at the beginning of measurement in the root zone, *Q_i_* [120], in a given time interval expressed as:

(1) $$\Delta S=Q_{o}-Q_{i}$$

(2) ET=P+I−R−D−ΔS

where Δ*S* shows the change in soil water storage over the 1 m soil profile, *P* is precipitation, *I* is irrigation, *R* is runoff and *D* is deep percolation, all expressed in millimetres. The term R was assumed to be negligible because the experiment was conducted on flat terrain and no high-intensity precipitation was experienced. In addition, irrigation was applied using a drip irrigation system and the rate of application did not surpass that of the soil infiltrability. A negative value for Δ*S* shows a decrease in the amount of water stored in the soil.

### 4.3. Soil Water Content Estimation

Soil water was measured weekly at 0.20 m intervals from the soil surface down to 1.00 m. Aluminium access tubes that protruded 0.10 m above soil level were covered to prevent rain or soil from entering. Maximum root depth was estimated from weekly measurements of soil water extraction with the neutron water meter model 503 DR CPN Hydroprobe (Campbell Pacific Nuclear, Martinez, CA, USA) that was calibrated for the experimental site. Root-zone soil water deficit calculations for irrigation were made over an assumed rooting depth of 1.00 m.

### 4.4. Model Calibration

Weather data, including daily minimum and maximum air temperature, as well as humidity, wind speed, incoming solar radiation and precipitation, were recorded by a nearby automated weather station. The automatic weather station consisted of an LI-200SA pyranometer (Li-Cor, Lincoln, NE, USA) to measure solar radiation, a cup anemometer (R.M. Young, Traverse, MI, USA) to measure average wind speed, a tipping bucket rain gauge (Texas Electronics, Dallas, TX, USA), an HMP60 relative humidity and temperature sensor (Vaisala, Woburn, MA, USA), and a CR200X datalogger (Campbell Scientific, Inc., Logan, UT, USA). For calibration of the SWB model, weather, soil and crop data collected in the field trial were used to determine crop-specific growth parameters for well-irrigated landrace, improved and hybrid pearl millet varieties for the 2018 growing season.

#### 4.4.1. VPD and SI Parameters

Vapour pressure deficit was calculated according to the following equation by Jovanovic, Annandale and Mhlauli [46]:

(3) $$\mathrm{VPD}=\left[\frac{e_{sTmax}\mp e_{sTmin}}{2}\right]-e_{a}$$

where *e_sTmax_* is saturated vapour pressure at maximum air temperature (Pa), *e_sTmin_* is saturated vapour pressure at minimum air temperature (Pa) and *e_a_* is actual vapour pressure (Pa). Saturated vapour pressure (*e_s_*) at maximum (*T_max_*) and minimum air temperature (*T_min_*) was calculated by replacing *T_a_* (actual air temperature) with *T_max_* and *T_min_* (°C) [119], as presented in Equation (4):

(4) $$e_{s}=0.611exp\left[\frac{17.27Ta}{Ta+237.3}\right]$$

*e_a_* (actual vapour pressure) was calculated from the measured daily *T_max_*, *T_min_, RH_max_* and *RH_min_*, using the following equation from Allen, Pereira, Raes and Smith [119]:

(5) $$e_{a}=\frac{e_{s}\left(T_{min}\right)\frac{RH_{max}}{100}+e_{s}\left(T_{max}\right)\frac{RH_{min}}{100}}{2}$$

As water becomes limiting, actual evapotranspiration will fall below the potential rate. A measure of the ratio of actual to reference evapotranspiration should therefore be an index of crop water status. Water stress occurs when available soil water does not meet potential transpiration demand, and it is calculated as the ratio of actual to potential transpiration. A water stress index can also be used to reduce the accumulation of growing day degrees, depending on phenology calculated with thermal time and modified by water stress. This dry matter is partitioned into roots, stems, leaves, grains, or fruits (Annandale, [49]). Such stress index (SI) is defined by Jackson et al. [121,122] as ranging from 0 (ample water) to 1 (maximum stress) and was computed from a soil water balance [86,123,124]:

(6) $$\mathrm{SI}=\frac{T}{\left({\mathrm{Fi}}_{\mathrm{transp}}PET\right)}$$

where SI is stress index, *T* is actual evapotranspiration (mm), and *PET* is potential evapotranspiration (mm).

#### 4.4.2. Radiation Limited Dry Matter Production

Many crop growth models are radiation driven and require an estimate of radiation use efficiency (RUE). In SWB, the radiation conversion efficiency is a crop-specific parameter used to calculate dry matter production under conditions of radiation limited growth, from Monteith [125], as follows:

(7) $$\mathrm{DM}=\mathrm{RUE}\overset{n}{\underset{i=1}{\sum }}{\mathrm{Fi}}_{\mathrm{RAD}}R_{s}$$

where DM is total dry matter production in kg m^−2^, which includes root mass. RUE is the slope of the regression between intercepted solar radiation and DM production, forced through the origin (kg MJ^−1^). RUE is also known as the radiation conversion efficiency (*E_c_*), FI_RAD_ is the fraction of daily value incremented each day (*i* = 1 to n) of solar radiation intercepted by the green, transpiring canopy and *R_s_* is the total fraction of daily value incremented each day (*i* = 1 to n) of incoming solar radiation in MJ m^−2^ day^−1^.

#### 4.4.3. Water Limited Dry Matter Production

Transpired water vapour leaves the plant by diffusion through stomata, and carbon dioxide is taken up for photosynthesis following the same pathway but in the opposite direction. The CO_2_ taken up is converted to organic carbon, and this process which is accompanied by transpirational water loss can be described by a simple gas exchange dry matter production model [126]. Transpiration limited growth is calculated using the relationship between dry matter accumulation and transpiration, from Tanner and Sinclair [127].

(8) $$\mathrm{DWR}=\frac{\left(\mathrm{DM}\times \mathrm{VPD}\right)}{\mathrm{ET}}$$

where, DWR (dry matter water ratio) is the water productivity [124] of the dry matter production per unit crop transpiration (*T*), adjusted to account for vapour pressure deficit (VPD) [127], and DM is total dry matter produced (kg m^−2^). In SWB, a simple gas exchange model that acknowledges the tight link between transpiration corrected for dry matter production and vapour pressure deficit (VPD) is used [44].

VPD is the seasonal average vapour pressure deficit. ET is the seasonal (from sowing to maturity) total evapotranspiration obtained using Equation (1). Both VPD and DWR are in Pa, while ET is in mm. Due to the difficulty in determining root dry matter, only top dry matter was measured, resulting in a smaller DWR than required in Equation (8). Similarly, transpiration (*T*) is difficult to measure, so ET was used, which also gives a lower DWR than the actual value. Evapotranspiration and top dry matter were therefore used to estimate a lower limit value of DWR. The DWR lower limit value was increased during model parameterisation until simulation data were observed in line with measured data. The estimation of lower limit DWR values was performed using measured top dry matter accumulation data at physiological maturity to avoid errors caused by plants losing leaves, as leaf senescence usually occurs after physiological maturity.

#### 4.4.4. Growing Degree Days

Growing degree days for the transition period between planting and emergence, vegetative and reproductive growth and leaf senescence were estimated by calibration against field measurements of phenology for all pearl millet varieties. The growing day degrees (GDD) (d °C) were calculated using the daily average air temperature, from Monteith [125]:

(9) $$\mathrm{GDD}=\overset{n}{\underset{i=1}{\sum }}\left(\frac{T_{max}+T_{min}}{2}-T_{b}\right)$$

where GDD is growing day degrees, Tmax is the maximum air temperature in °C, Tmin is the minimum air temperature in °C, *T_b_* is the base temperature in °C, *n* is the number of days and *i* is the daily increment until n days. The base temperature for calculating GDD is the minimum threshold temperature below which plant development ceases. Growing degree days were accumulated from the date of sowing to maturity of each pearl millet cultivar. In this research, a *T_b_* value of 10 °C and a maximum temperature of 45 °C were chosen for pearl millet [67]. If the daily average temperature was less than the base temperature, then the GDD value was assumed equal to zero [84]. GDD was used to estimate the duration of the different crop development stages. Growing day degrees for emergence were computed as the difference between average daily temperature and base temperature, starting from sowing date to emergence.

#### 4.4.5. Radiation Interception

A Decagon Sunfleck ceptometer (Decagon Devices, Pullman, WA, USA) was used to measured fractional interception of photosynthetically active radiation on a weekly basis. On clear-sky days, measurements were performed between 10:00 and 12:00. Five reference values above and five readings below the canopy were averaged in a series of measurements. FI_PAR_ (fractional interception of photosynthetic active radiation) was determined using the following formula:

(10) $${\mathrm{FI}}_{\mathrm{PAR}}=\left[1-\frac{\mathrm{PAR}\;\mathrm{below}\;\mathrm{canopy}}{\mathrm{PAR}\;\mathrm{above}\;\mathrm{canopy}}\right]$$

The Beer–Bouguer law was used to determine the canopy extinction coefficient for PAR [128]:

(11) $${\mathrm{FI}}_{\mathrm{PAR}}=1-e^{\left(-K_{\mathrm{PAR}}\times \mathrm{LAI}\right)}$$

The canopy extinction coefficient for PAR is represented by K_PAR_. Field measurements of LAI and FI_PAR_ were used to calculate K_PAR_ values. Dry matter production can be estimated using K_PAR_ as a function of intercepted PAR. SWB requires the canopy extinction coefficient for solar radiation (K_s_), as fractional interception of solar radiation (FI_RAD_) is used to predict radiation limited dry matter production [125], and for partitioning evapotranspiration into evaporation from the soil surface and crop transpiration [129]. The procedure recommended by Campbell and Van Evert [128] was used to convert K_PAR_ into K_s_:

(12) $$K_{s}=K_{\mathrm{bd}}\sqrt{a_{s}}$$

(13) $$K_{\mathrm{bd}}=\frac{K_{\mathrm{PAR}}}{\sqrt{a_{p}}}$$

(14) $$a_{s}=\sqrt{a_{p}a_{n}}$$

where K_bd_ is the canopy radiation extinction coefficient for black leaves and diffuse radiation, a_p_ is the absorptance of PAR, and a_s_ is leaf absorptance of near-infrared radiation (NIR, 0.7–3 m). The value of a_p_ was set to 0.8, while the value of a_n_ was set to 0.2 [130]. The geometric mean of the absorptances in the PAR and NIR spectrums is referred to as a_s_.

#### 4.4.6. Above-Ground Biomass Partitioning

In SWB, the daily increment in DM is computed to be either limited by transpiration or limited by radiation [56], with the partitioning of assimilates among different plant organs influenced by plant water status. Specific leaf area (SLA) was obtained as the total leaf area (LA) divided by the dry mass of leaves per sampling area.

(15) $$\mathrm{Specific}\;\mathrm{leaf}\;\mathrm{area}\;\left(\mathrm{SLA}\right)=\frac{\mathrm{LA}}{\mathrm{LDM}}$$

The SLA, LAI, and canopy dry matter (CDM) were used to calculate the leaf-stem partitioning parameter (p) [46]. The leaf-stem partitioning parameter is represented as the slope of the regression line between CDM and (SLA.CDM)/LAI—1 in m^2^ kg^−1^. LAI is the leaf area index (m^2^ m^−2^), and SLA is the specific leaf area (m^2^ kg^−1^). SDM is stem dry matter.

(16) $$\mathrm{LDM}=\mathrm{CDM}/\left(1+\mathrm{pCDM}\right)$$

(17) SDM=CDM−LDM

Grain yield, LDM, CDM and SDM are all measured in kg m^−2^.

### 4.5. Data Collection for Model Calibration

For SWB model calibration the following crop growth parameters for each of the crops were recorded: canopy extinction coefficient (Ks) for total solar radiation, dry matter: transpiration ratio, adjusted for vapour pressure deficit (DWR, kg·kg^−1^·Pa), radiation use efficiency (RUE, kg·MJ^−1^), leaf-stem dry matter partitioning (p, m^2^·kg^−1^) and specific leaf area (SLA, m^2^·kg^−1^).

### 4.6. Model Validation

A three-year dryland field experiment was established to investigate crop response to dry spells at the Hatfield Experimental Farm of the University of Pretoria in Pretoria, South Africa, with planting dates in February 2016, February 2017 and December 2018. Three pearl millet varieties with contrasting agronomic characteristics, namely Kantana, Kangara and Agrigreen, were grown. Kantana is a landrace variety with a longer duration to reach maturity, high plant height and a greater biomass. Landraces have high adaptation to prevalent abiotic stresses. Kangara is an improved early maturity variety released in Namibia in 1998 [6], characterised by high grain yield under rainfed conditions. Agrigreen is a high-yielding hybrid variety, predominantly grown as a fodder crop but is also valued for its grain. Hybrids are developed due to their yield superiority over open pollinated varieties; however, they are often outperformed by landrace and improved varieties under rainfed conditions [131].

These pearl millet varieties were subjected to three different irrigation regimes (rainfed, irrigated every week and every second week to field capacity). The soil was similar to that used to parameterise the model. The experiment was set up in a three-replication randomised complete block. Pearl millet was planted at a 0.45 m inter-row spacing with a 0.14 m intra-row spacing, in plots measuring 4.95 m by 9.5 m. A calibrated neutron probe was used to measure the amount of water in the soil on a weekly basis. Drip irrigation was used to irrigate the plots. The rainfed treatments and every second week irrigated treatments used to validate the model were as follows:

1. Irrigated weekly to field capacity until the end of the growing season.
2. Irrigated to field capacity on a fortnightly basis until the end of the growing season.
3. Rainfed (dryland) until the end of the growing season.

## 5. Conclusions

All the varieties had almost similar water use efficiency (for grain yield), radiation use efficiency and degree days to emergence. The specific leaf area, canopy extinction coefficient for total solar radiation and flowering day degrees parameters were different among the varieties. The hybrid variety portrayed a determinate growth habit; therefore, if similar varieties grow in this way, hybrid parameters must be used. However, if the variety is late maturing and indeterminate, modellers are advised to use landrace parameters. If the modellers have an early maturity variety that is indeterminate, then the improved variety parameters should be used. The average values for canopy extinction coefficient for photosynthetic active radiation differ largely among the three varieties; therefore, using average values across the varieties is not advisable. The parameters need to be used with the model to evaluate results. Despite the lack of actual long-term yield data to compare to simulations, the authors are confident that the calibrated SWB model will be useful in estimating pearl millet yields under various production conditions. It is, however, recommended that crop parameters should also be determined for other pearl millet varieties to make the SWB model more useful for the management of irrigation, long-term yield projection and planning purposes.

## Figures and Tables

**Table 1:** Weekly soil water balance components (mm) for three pearl millet varieties (December–April of 2017/2018). Precipitation (P) and irrigation (I), evapotranspiration (ET), drainage (D), runoff (R), amount of water contents at the beginning of a measurement period in the root zone (Q_o_), amount of water contents at the end of a measurement period in the root zone (Q_i_) and change in water storage (ΔS).

Landrace Pearl Millet (Kantana)									Improved Pearl Millet (Kangara)							Hybrid (Agrigreen)						
Date	P	I	ET	D	R	Q_i_	Q_o_	∆S	I	ET	D	R	Q_i_	Q_o_	ΔS	I	ET	D	R	Q_i_	Q_o_	ΔS
14 December 2017	26	0	13	0	0	315	328	−14	0	14	0	0	264	276	−13	0	12	0	0	292	306	−14
22 December 2017	28	0	7	0	0	328	350	−21	28	8	0	0	276	324	−48	0	7	0	0	306	327	−21
29 December 2017	15	0	13	0	0	350	352	−2	4	12	0	0	324	332	−8	0	18	1	0	327	322	4
5 January 2018	7	10	29	0	0	352	340	12	8	23	0	0	332	324	8	0	38	1	0	322	290	32
12 January 2018	11	23	50	0	0	340	323	17	31	47	0	0	324	319	5	40	50	0	0	290	291	−1
19 January 2018	26	46	55	0	0	323	339	−17	40	54	0	0	319	332	−12	51	54	0	0	291	314	−23
31 January 2018	34	39	81	0	0	339	331	8	72	81	0	0	332	356	−25	71	81	8	0	314	330	−16
5 February 2018	12	37	45	0	0	331	336	−4	50	36	0	0	356	383	−27	60	36	4	0	330	363	−32
18 February 2018	79	46	87	28	0	336	345	−10	0	87	19	0	383	355	27	42	89	28	0	363	366	−3
5 March 2018	15	60	81	6	0	345	334	12	81	81	7	0	355	363	−8	66	84	6	0	366	357	9
10 March 2018	3	31	31	1	0	334	336	−2	63	31	0	0	363	398	−34	52	31	1	0	357	380	−23
16 March 2018	2	59	40	1	0	336	357	−21	60	40	1	0	398	420	−22	52	39	1	0	380	395	−14
24 March 2018	209	0	44	88	0	357	434	−77	0	44	64	0	420	521	−101	0	45	51	0	395	508	−113
2 April 2018	8	38	40	34	0	434	406	29	0	41	53	0	521	435	86	24	41	29	0	508	470	38
8 April 2018	1	16	28	3	0	406	391	14								0	28	3	0	470	440	30
14 April 2018	64	0	25	17	0	391	413	−22														

**Table 2:** Seasonal soil water balance (mm) for three pearl millet varieties (December–April of 2017/2018). Precipitation (P) and irrigation (I), evapotranspiration (ET), drainage (D), runoff (R), evapotranspiration (ET_o_) and average vapour pressure deficit is in Pascal (Pa) (Avg. VPD).

Landrace Pearl Millet (Kantana)							Improved Pearl Millet (Kantana)						Hybrid (Agrigreen)					
P	I	ET	D	R	ETo	AvgVPD	I	ET	D	R	ETo	AvgVPD	I	ET	D	R	ETo	AvgVPD
542	405	670	179	0	436	0.78	437	598	144	0	412	0.83	458	653	134	0	425	0.81

**Table 4:** Treatments in 2016, 2017 and 2017/2018 varieties (V_1_, V_2_, V_3_) and water irrigation regimes (I_0_, I_1_, I_2_).

Pearl Millet Varieties	Irrigation Regime		
	I_0_	I_1_	I_2_
V_1_	0	1	2
V_2_	0	1	2
V_3_	0	1	2

Note: V_1_ = hybrid (Agrigreen), V_2_ = open pollinated landrace (Kantana), V_3_ = open pollinated improved (Kangara).

## Data Availability

Details regarding data availability will be provided during review.
